# Metabolic function-based normalization improves transcriptome data-driven reduction of genome-scale metabolic models

**DOI:** 10.1038/s41540-023-00281-w

**Published:** 2023-05-20

**Authors:** Mahdi Jalili, Martin Scharm, Olaf Wolkenhauer, Ali Salehzadeh-Yazdi

**Affiliations:** 1 Hematology, Oncology and SCT Research Center, Tehran University of Medical Sciences, Tehran, Iran; 2 CODE AHOI, Rostock, Germany; 3 Department of Systems Biology and Bioinformatics, University of Rostock, Rostock, Germany; 4 Stellenbosch University, Stellenbosch Institute for Advanced Study (STIAS), Wallenberg Research Centre, Stellenbosch, South Africa; 5 Leibniz Institute for Food Systems Biology at the Technical University Munich, Freising, Germany; 6 School of Science, Constructor University, Bremen, Germany

**Keywords:** Biochemical networks, Metabolic engineering

## Abstract

Genome-scale metabolic models (GEMs) are extensively used to simulate cell metabolism and predict cell phenotypes. GEMs can also be tailored to generate context-specific GEMs, using omics data integration approaches. To date, many integration approaches have been developed, however, each with specific pros and cons; and none of these algorithms systematically outperforms the others. The key to successful implementation of such integration algorithms lies in the optimal selection of parameters, and thresholding is a crucial component in this process. To improve the predictive accuracy of context-specific models, we introduce a new integration framework that improves the ranking of related genes and homogenizes the expression values of those gene sets using single-sample Gene Set Enrichment Analysis (ssGSEA). In this study, we coupled ssGSEA with GIMME and validated the advantages of the proposed framework to predict the ethanol formation of yeast grown in the glucose-limited chemostats, and to simulate metabolic behaviors of yeast growth in four different carbon sources. This framework enhances the predictive accuracy of GIMME which we demonstrate for predicting the yeast physiology in nutrient-limited cultures.

## Introduction

To illustrate the genotype-phenotype relationship of metabolic phenotypes in an environment, we require a map of genome-scale biochemical reactions and their comprehensive connections. The GEnome-scale Metabolic models (GEM) provide a mathematical framework to gain understanding into metabolic capacity of a cell: enable system-wide analysis of genetic perturbations and metabolic engineering, identify the constraints that the chemical interactions operate under, explore metabolic diseases, as well as to find essential enzymatic reactions^1^.

Following the introduction of GEMs, a new challenge arose: the integration of omics data into a GEM for a better prediction of the metabolic functionalities. The conjunction of gene expression data and GEMs leads to a deeper understanding of the occurrence of certain changes in different conditions and creates condition- and context-specific models^2^. Since Covert et al. presented the first conceptual framework on transcriptional regulation of metabolic models in 2001^3^, several approaches have been developed to investigate how the integration of gene expression could affect the content and predictive accuracy of a GEM^4–6^. These frameworks, which either are based on a new algorithm or are modifications of the previous frameworks, differ in their assumptions and mathematical formulations. All currently available integration methods are listed in Table 1. With the increasing interest in these integration approaches, multiple publications have also focused on the benchmarking of the methods used to generate context-specific models, and evaluated the advantages and disadvantages of different approaches^2,7^. Currently, none of these algorithms systematically outperforms the others, as each of them has specific pros and cons depending on the type of data available to tailor the GEM^2^.

**Table 1:** Summary of developed integration algorithms to generate context-specific GEMs.

Algorithm	Year	Organism	Description	Programming language
Covert-01^3^	2001	—	Introduction of a theoretical framework to broaden the predictive capabilities of GEMs with the incorporation of a transcriptional regulatory network using Boolean logic formalism.	NA
Covert-02^26^	2002	*E. coli*	The previous framework, applied to the central metabolism in *E.coli*.	NA
Akesson-04^27^	2004	*S. cerevisiae*	Introduction of a theoretical framework for the integration of transcriptome data into metabolic models. In this framework, the flux of some reactions constraint to zero, if their associated genes are not expressed.	NA
SR‐FBA^28^	2007	*E. coli*	SR-FBA uses the same framework as Covert-01, but with a different formulation. SR-FBA identifies genes and metabolic functions in which regulation is not optimally tuned for cellular flux demands.	NA
Shlomi-08^17^	2008	*H. sapiens, and S. cerevisiae*	The integrative metabolic analysis (iMAT) framework modified the Boolean mapping for tri-valued gene expression levels; high, low, and moderately expressed genes.	NA
GIMME^9^	2008	*E. coli, and H. sapiens*	GIMME presented a conceptual framework that indicates how consistent a set of gene expression data is with a desired metabolic objective.	MATLAB + COBRA
E-Flux^29^	2009	*M. tuberculosis*	In contrast to previously developed methods for metabolically interpreting gene expression data, E-Flux uses the normalized gene expression level to approximate and constrain the maximum flux of corresponding reactions.	MATLAB + COBRA
Moxley^30^	2009	*S. cerevisiae*	In this framework, the developers considered the correlation between changes in gene expression and reaction levels as well as topological parameters of the metabolic networks.	NA
MBA^31^	2010	*H. sapiens*	This framework has been developed to integrate a variety of omics data as well as literature‐based knowledge to generate context-specific models. Accordingly, the algorithm takes two reaction sets as input (extracted from omics data analysis and literature) and reconstructs a model containing as many as possible of input reactions, and a minimal set of reactions that are required for model consistency.	MATLAB + COBRA
MADE^32^	2011	*S. cerevisiae*	Selecting arbitrary thresholds to find active/deactive reactions was the main challenge in previously developed algorithms. MADE tackles this problem by comparing gene expression levels across multiple conditions to identify activation/deactivation patterns.	MATLAB + COBRA
tFBA^33^	2011	*S. cerevisiae*	tFBA uses the same framework as MADE, but with a different formulation. Basically, tFBA assumes that if the activity of a gene in two different conditions drastically changes, the associated flux will change accordingly.	NA
RELATCH^34^	2012	*E. coli, S. cerevisiae, and B. subtilis*	RELATCH defined a “relative optimality” conceptual framework to create a context-specific model. In this framework, perturbed cells preserve a relative metabolic flux pattern from a reference state using metabolic adaptation and regulatory reprogramming.	MATLAB + COBRA
INIT^16^	2012	*H. sapiens*	INIT integrates proteomic or transcriptomic data into a GEM. The INIT algorithm maximizes the activation of certain reactions associated with highly expressed genes while minimizing the utilization of reactions associated with absent proteins.	MATLAB + COBRA + RAVEN
mCADRE^35^	2012	*H. sapiens*	mCARDE uses the same framework as MBA, but with a different formulation for non-core reactions. mCARDE ranks non-core reactions according to their expression as well as weighted connectivity in the given network, and then keeps reactions which are required for model consistency.	MATLAB + COBRA
AdaM^36^	2012	*E. coli*	AdaM provides a framework to evaluate adaptation upon external perturbation. AdaM extracts minimal operating networks (to characterize the transitional behavior) by integration of time-series transcriptomics data with flux-based bi-level optimization.	NA
Lee-12^37^	2012	*S. cerevisiae*	In this theoretical framework, the objective function of a context-specific model is justified by gene expression data, while the flux constraints of the model remain untouched.	MATLAB + COBRA
Fang-12^38^	2012	*M. tuberculosis*	This approach works according to a) relative gene expression between a reference state and a perturbed condition, b) the correlation between gene transcription levels and enzymatic activity, c) the justification of the biomass composition for the perturbed state.	MATLAB + COBRA
GX–FBA^39^	2012	*Y. pestis, and S. cerevisiae*	GX-FBA optimizes the pattern of hierarchical regulation, as well as the level of differential gene-expression within the framework of metabolic constraints.	MATLAB + COBRA
TEAM^40^	2012	*S. oneidensis*	TEAM uses the same framework as GIMME, but differs in that it takes advantage of large compendium of gene expression data to estimate each gene’s unique transcriptional signature.	NA
GIM3E^41^	2013	*S. typhimurium*	GIM^3^E uses the same framework as GIMME, but in this algorithm, the estimated penalties minimize reactions that have weaker supporting evidence.	Python+COBRApy
EXAMO^42^	2013	*S. cerevisiae*	EXAMO uses the same framework as iMAT, but with a different formulation. EXAMO considers high-frequency reactions (HFR) as active reactions and then minimizes the given GEM so that all HFR should be able to carry flux.	Python standalone
MTA^43^	2013	*E. coli*, *S. cerevisiae, M. musculus, and H. sapiens*	MTA uses the same integration framework as iMAT, with different application for drug target prediction. In fact, MTA identifies drug targets that alter the metabolism in order to retrieve it back to the initial healthy state.	NA
FASTCORE^44^	2014	*H. sapiens*	FASTCORE uses the same framework as MBA, but with a different formulation. Accordingly, FASTCORE takes a core set of active reactions as input, and then searches for a flux consistent subnetwork.	MATLAB + COBRA
tINIT^45^	2014	*H. sapiens*	INIT-based GEMs are not functional models and could not be used for simulations. Therefore, tINIT developers tackled this issue by defining metabolic tasks, which the resulting model should be able to perform.	NA
E-Fmin^46^	2014	*E. coli, and S. cerevisiae*	E-Fmin is a modified extension of GIMME which forces biomass production to carry non-zero flux, whereas GIMME requires certain metabolic functionalities to be active above condition-dependent thresholds.	MATLAB
METRADE^47^	2015	*E. coli*	METRADE developed a multi-omic framework that integrates gene expression and codon usage into the GEM, and uses a multi-objective optimization algorithm to optimize metabolic phenotypes.	MATLAB
Lsei-FBA^48^	2015	*H. sapiens*	Lsei-FBA’s developers argued that the performance of existing methods was optimized for large changes in gene expression. To tackle this issue, Lsei-FBA uses the fold changes in mRNA gene expression to estimate the changes in the metabolic network.	R-package
FASTCORMICS^49^	2015	*H. sapiens*	FASTCORMICS is adapted FASTCORE for the direct integration of microarray data.	MATLAB + COBRA
TREM-Flux^50^	2015	*C. reinhardtii*	This algorithm integrates time-resolved metabolomics and transcriptomics data (performed based on E-Flux method).	MATLAB
RegrEx^51^	2015	*H. sapiens*	RegrEx is a fully automated framework that extracts a context-specific model by maximizing the correlation of flux distribution and a given data.	MATLAB + COBRA
CORDA^52^	2016	*H. sapiens*	CORDA is based on a dependency assessment approach which identifies the dependency of desirable reactions (i.e. reactions with high experimental evidence) on undesirable reactions (i.e. reactions with no experimental evidence).	MATLAB + COBRA
OM-FBA^53^	2016	*S. cerevisiae*	OM-FBA uses the same framework as Lee-12, but it deploys a “Phenotype Match” algorithm to derive an objective function to be correlated with the transcriptomics data via regression analysis.	MATLAB
E-Flux2^54^	2016	*E. coli, and S. cerevisiae*	E-Flux2 is an extension of E-Flux which is combined with minimization of *l*^*2*^ norm.	MATLAB+Java+ (MOST)
SPOT^54^	2016	*E. coli, and S. cerevisiae*	SPOT uses pearson correlation with transcriptomic data that can be combined with carbon source or objective function.	MATLAB+Java+ (MOST)
metaboGSE^55^	2018	*Y. lipolytica, and M. musculus*	metaboGSE introduced a new framework of creating condition-specific models. This algorithm creates a series of metabolic sub-networks by removing genes from a GEM. Then, sub-networks are evaluated via a fitness function.	R-package
Benchmark-driven^56^	2019	*H. sapiens*	Benchmark-driven approach is the last developed framework that is devised based on the advantageous features and bottlenecks of the selected algorithms.	MATLAB

One of the main challenges that limits overall predictive capability of these methods is setting different parameters e.g., gene expression threshold to find significantly differential expressed genes^7^. As enzymes have different expression levels and activities, determination of whether the protein is expressed or not by applying a uniform threshold for all expression data is suboptimal^8^. Here, we propose tackling this problem by transforming the transcription data to a higher-level space (gene sets instead of genes) which is a more biologically relevant set of features, which then can be integrated into a GEM. Critical for such transformation is the selection of the annotation set used to classify genes to respective gene groups: here we used a high-quality, manually derived set of annotations, which comprised mainly metabolic proteins. We therefore combined existing methods for transcriptional data integration into a GEM with a data normalization routine in order to more accurately predict the metabolic phenotypes of a model organism *Saccharomyces cerevisiae*. In general, our framework can be combined with all developed integration methods, however, here we illustrate our approach using the Gene Inactivity Moderated by Metabolism and Expression (GIMME)^9^ algorithm.

We first analyzed the transcription data by ssGSEA, an extension of Gene Set Enrichment Analysis (GSEA)^10^, to calculate enrichment scores (ES) for each annotated gene set which associated to the particular biological processes or pathways. Actually, ES represents the degree and ranks the genes in a particular gene set according to the values of expression. Thus, we reconciled the enrichment score with the GIMME algorithm. We show that the models, generated using normalized expression data, outperform the accuracy of standard GIMME models in certain cases.

In this study, the predictive accuracy of context-specific metabolic models was evaluated using their ability to predict metabolic fluxes under different scenarios. The prediction accuracy was determined by comparing the predicted flux values with experimentally measured values and was evaluated using statistical metrics. Additionally, the accuracy of essential gene prediction, growth rates and other phenotypic traits under different environmental conditions could also be taken into consideration.

## Results

### Predicting growth in glucose-limited chemostats

We evaluated and compared context-specific GEMs of *S. cerevisiae* built by different methods. First, we assessed their performance using the experimental data from glucose-limited chemostats at varying dilution rates. For each medium dilution rate of the chemostat, we generated respective context-specific models (using both GIMME and the combined method, ssGSEA-GIMME) using the respective RNA-seq data. Then we benchmarked the performance of these methods in predicting both intracellular and extracellular metabolic fluxes, based on the experimentally determined fluxes of glucose and maximal oxygen consumption by *S. cerevisiae* cultures.

**Fig. 1:**
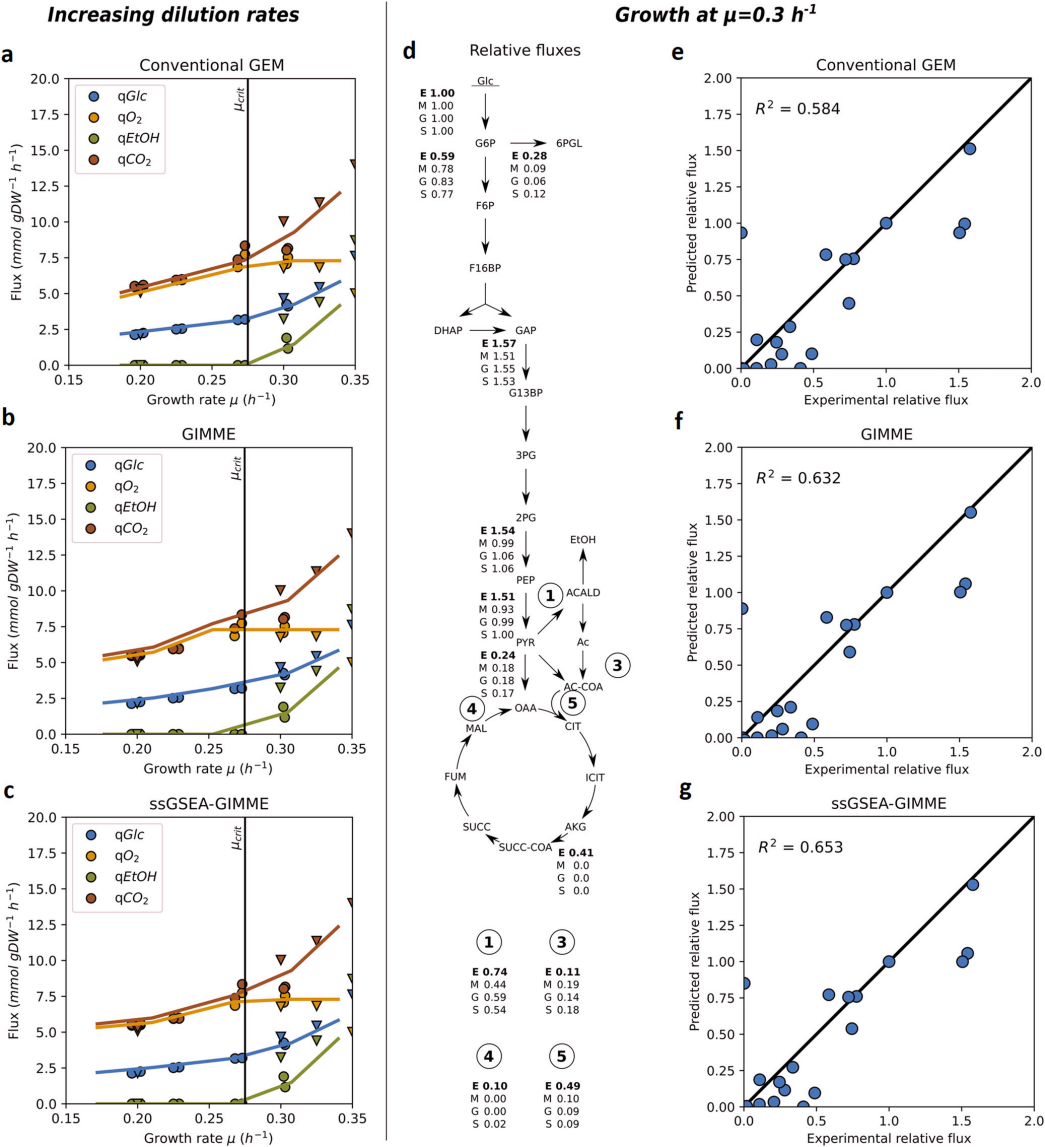
Characterization of the predictions of context-specific models for growth of *S. cerevisiae* under glucose-limited conditions. **a**–**c** Flux predictions of metabolite exchange (glucose, oxygen, ethanol and carbon dioxide) for changing dilution rates for glucose-limited chemostats by the conventional Yeast8.4.2 **a** and the context-specific models, created using GIMME **b** and ssGSEA-GIMME **c**. Experimental data, triangles taken from ref. ^12^ and circles from ref. ^22^. **d** Comparison of experimentally determined metabolic fluxes of the central carbon metabolism (E) and predictions by the conventional GEM (M), GIMME (G) and ssGSEA-GIMME (S) model at *μ* = 0.30 h^-1^. **e**–**g** Correlation between the experimental data (X-axis) and model predictions (Y-axis) for different model implementations. Experimental data in **d** and on the X-axes of **e**–**g** taken from ref. ^57^. Flux values were normalized to that of hexokinase reaction (*v*_*HEX*_^*rel*^ = 1). Glc glucose, Mal maltose, G6P glucose 6-phosphate, F6P fructose 6-phosphate, 6PGL 6-phosphogluconolactone, F16BP fructose 1,6-bisphosphate, DHAP dihydroxyacetone phosphate, GAP glycerol aldehyde 3-phosphate, G13BP 1,3-bisphosphoglycerate, 3PG 3-phosphoglycerate, 2PG 2-phosphoglycerate, PEP phosphoenolpyruvate, PYR pyruvate, ACALD acetaldehyde, EtOH ethanol, Ac acetate, AC-COA acetyl-CoA, OAA oxaloacetate, CIT citrate, ICIT, isocitrate; AKG, alpha-ketoglutarate; SUCC-COA, succinyl-CoA; SUCC, succinate; FUM, fumarate; MAL, malate; GLX, glyoxylate.

We first compared the flux balance analysis (FBA) predictions of the main exchange fluxes for both the conventional Yeast8.4.2 and the context-specific models (Fig. 1a–c). All three methods correctly predicted fluxes in the range of growth rate $\mu \,<\, 0.25{h}^{-1}$, as the growth in this range is limited only by the substrate availability. We also checked the flux intervals that would result in the same value of the objective function (proxy for the specific growth rate) using flux variability analysis (FVA). However, we noticed only minor changes in the sums of flux variability intervals when comparing GIMME and ssGSEA-GIMME.

Under glucose-limited conditions, *S. cerevisiae* exhibits fully-respiratory energy harvesting until a certain growth rate, the so-called critical growth rate ${\mu }_{{crit}}$, is reached. For growth with rates above ${\mu }_{{crit}}$, ethanol formation is detected. For the wild-type *S. cerevisiae*, the value of ${\mu }_{{crit}}=0.28{h}^{-1}$ for glucose-limited chemostats was determined by a number of studies^11,12^. As expected, both the conventional GEM and context-specific models predicted ethanol formation above the ${\mu }_{{crit}}$, with predicted values of ${\mu }_{{crit}}=0.273\,{h}^{-1}$ for the conventional GEM, and ${\mu }_{{crit}}=0.253\,{h}^{-1}$ and ${\mu }_{{crit}}=0.272\,{h}^{-1}$ for GIMME and ssGSEA-GIMME, respectively. From the modeling perspective, it was shown that for this to happen, a second global constraint (the first being the limitation of the substrate, and the second being limited oxygen uptake) has to be defined in the model (see^13^ for more details). Yet GIMME predicted ethanol formation at a lower growth rate than the experimental data suggested. Meanwhile, both the conventional GEM and ssGSEA-GIMME captured the onset of ethanol formation correctly, in agreement to the experimentally determined critical growth rate.

Furthermore, we compared the predictions of intracellular fluxes at $\mu =0.30{h}^{-1}$ for all three model implementations with previously published literature data (Fig. 1d). Unsurprisingly, when taking into account all the flux predictions in the metabolic network, both context-specific models showed moderately improved predictions compared to the conventional GEM (Fig. 1e–g). Both GIMME and ssGSEA-GIMME models showed rather similar flux profiles (Fig. 1f, g), in agreement with the similar exometabolite flux predictions in Fig. 1b, c. Taking together the results of these two tests, we suggest that ssGSEA-GIMME indeed has potential to refine the predictions of metabolic fluxes better, compared to GIMME. Importantly, ssGSEA-GIMME performed better in predicting the critical growth rate of ethanol formation for glucose-limited cultures of *S. cerevisiae*.

### Testing flux predictions of growth on different substrates

We achieved only moderate improvement on predicting growth on glucose-limited chemostat cultures with ssGSEA-GIMME, compared to GIMME alone. Therefore, we wanted to determine whether the improvement (or lack thereof) of flux predictions depends on the growth condition (nutrients supplied). To that end, we further explored the predictive capabilities of the context-specific models by using the previously published data^14^. In the study, *S. cerevisiae* was grown in four different carbon source-limited chemostats (glucose-, maltose-, ethanol-, and acetate-limited cultures) at the dilution rate $D=0.1{h}^{-1}$.

**Fig. 2:**
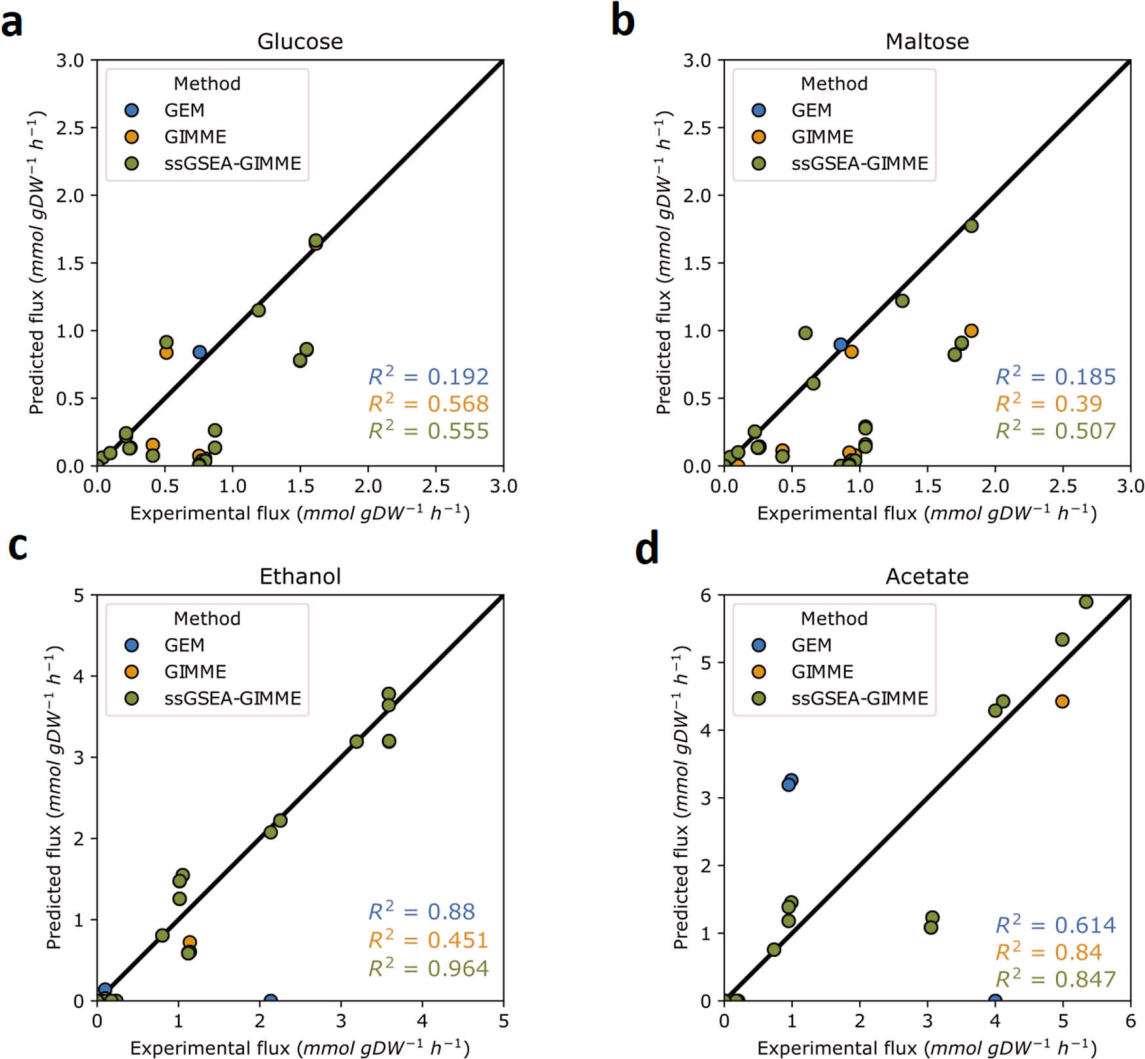
Comparison of flux predictions for carbon-limited chemostat cultures with glucose, maltose ethanol or acetate as the single carbon source. Experimental data on X-axis taken from ref. ^14^.

Evaluation of the determination coefficient for the predictions of the computational methods (Fig. 2) suggested that, compared to the conventional GEM, GIMME alone improved predictions for all carbon sources but ethanol. For growth on glucose and acetate, the context-specific models, with and without applying ssGSEA, showed very similar predictions. We saw similar effects when taking into consideration the fluxes in central carbon metabolism (Fig. 2; see Figure S1 for more details).

he low accuracy of the conventional GEM for predicting glucose-limited growth (Fig. 2a) was, among other fluxes, due to unreasonably high fluxes through succinate dehydrogenase, surpassing the experimentally determined value by some 6-fold (Figure S1). Contrary to the conventional GEM, both GIMME and ssGSEA-GIMME models showed improved predictions for the flux through succinate dehydrogenase.

Both GIMME and ssGSEA-GIMME showed incremental improvement in predicting fluxes for growth on maltose and acetate (Fig. 2b). For growth on ethanol, only ssGSEA-GIMME showed improvement of the flux predictions, compared to the conventional GEM. To our surprise, predictions of the model, generated with GIMME alone were worse than these of the conventional GEM. Based on our observations, here we conclude that the improvement of context-specific models by data normalization indeed is dependent on the condition analyzed. While normalization of the expression data, using, among other methods, ssGSEA, remarkably improves the predictions for some growth conditions, prediction of quantitatively-sound flux distributions are still very cumbersome for some growth conditions.

## Discussion

Since 2001, different frameworks have been developed or modified to investigate how gene expression integration into GEM could influence model content and increase its predictive accuracy.

Integrating gene expression into fluxes is intrinsically hindered by the assumption that “the mRNA transcript level is correlated with enzyme activity”, which should be a systemic property of metabolism. In addition, thresholding is a crucial step in gene expression analysis, as it helps to identify and filter out genes that are not differentially expressed, thus reducing the amount of noise in the data. There are different types of thresholding methods, such as standard deviation thresholding (STANDEP) and local T2, which have been used in gene expression analysis. However, these thresholding approaches have limitations, particularly when it comes to detecting subtle changes in gene expression, especially in complex systems such as metabolic networks.

**Fig. 3:**
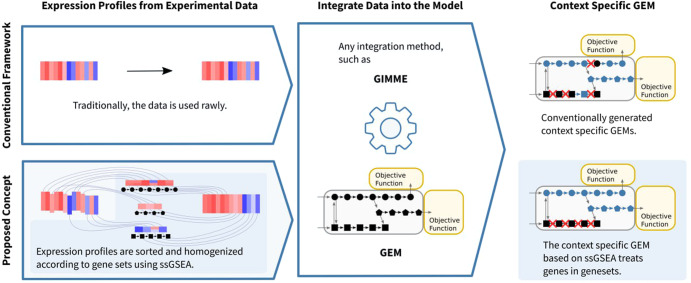
Workflow of the proposed concept in contrast to the standard framework. Circle, square and hexagon symbols stand for a given pathway. Here, we depicted that in the traditional (existing) framework a whole pathway flux might be inhibited due to a single enzyme down-regulation.

Therefore, here we retrieve a functional profile of the gene sets (a systemic mode), in order to better integrate the underlying metabolic processes. Our central hypothesis is that gene set enrichment as a pre-processing step can increase predictive accuracy of context-specific models. This approach differs from traditional thresholding methods in that it focuses on the functional enrichment of gene sets rather than individual genes, making it more robust in detecting subtle changes in gene expression. ssGSEA works by normalizing gene expression data based on the gene’s metabolic function, which allows for a more accurate representation of the underlying biological processes (Fig. 3).

To evaluate this proposed framework, we combined ssGSEA with the GIMME algorithm and applied it to two gene expression datasets; yeast grown in glucose-limited chemostats at different dilution rates and growth of *S. cerevisiae* in four different carbon source-limited chemostats at a constant dilution rate. This is a first attempt to integrate normalized (based on metabolic function) gene expression data into GEM, however, the framework could be improved by considering other enrichment analysis approaches^15^. However, our attempts to apply to generate context-specific models using INIT^16^ or iMAT^17^ resulted in models unable to predict biomass formation. While this issue could be mitigated by supervised generation of context-specific models (i.e. manually defining the reactions which should not be removed during generation), such a need severely hampers both usability and usefulness of models generated by these methods.

Our assessment of the predictions by GIMME and ssGSEA-GIMME shows that the combined framework performs well under all scenarios, outperforms conventional GIMME in some scenarios, and shows promise in its ability to improve the performance of integration approaches.

Conclusively, our findings suggest that ssGSEA has the potential to improve the accuracy of metabolic models, particularly when integrated with the GIMME algorithm. While this is a novel idea and requires further validation with more data, it highlights the importance of considering functional enrichment in gene expression analysis, and the potential benefits of using a method like ssGSEA.

While our approach has shown promising results, it is important to acknowledge some of the limitations of our study.

First, the sample size in our analysis was limited; therefore, further validation on larger sample sizes is necessary to confirm the robustness of our findings. Additionally, we were limited in our choice of data sources, and further research could explore how our approach performs with a broader range of data.

Second, the importance of carefully considering the impact of thresholding on the accuracy of context-specific metabolic models. The integration of transcriptomic data into a genome-scale metabolic model plays a critical role in studying cellular metabolism and developing context-specific metabolic models. The thresholding of gene expression data significantly impacts the accuracy of these models. Thresholding refers to setting a minimum threshold for gene expression levels and determining which genes are included or excluded from the models. This process is used to reduce the impact of noise in the data and to increase the accuracy of the models. However, the impact of thresholding on the accuracy of context-specific metabolic models is a complex issue that requires a comprehensive understanding of the interplay between gene expression data and metabolic pathways.

Three key papers in this field include “StanDep: Capturing transcriptomic variability improves context-specific metabolic models” by Joshi et al.^18^, “Assessing key decisions for transcriptomic data integration in biochemical networks” by Richelle et al.^19^, and “Guidelines for extracting biologically relevant context-specific metabolic models using gene expression data” by Gopalakrishnan et al.^20^. These papers provide valuable insights into the best practices for integrating transcriptomic data into metabolic models and the impact of thresholding on their accuracy.

The ssGSEA-based method that we developed represents a new approach to investigating the impact of thresholding on the accuracy of context-specific models. Our method is distinct from other approaches in the field, as it utilizes ssGSEA to extract biologically relevant information from gene expression data. This innovative approach brings several benefits to the field, including improved accuracy and efficiency, as demonstrated by our results.

Additionally, the core reaction sets generated using ssGSEA may differ from those generated using other local thresholding approaches. This difference may arise due to the different criteria used by ssGSEA to identify gene sets for reduction. As for handling lowly expressed genes with a low-flux metabolic activity, our method does not inherently exclude these genes from the final model. Instead, the model reduction process performed by ssGSEA focuses on the metabolic function of genes and reactions as well as gene ontology rather than their expression level or flux activity. In this way, the reduction process can capture the most relevant metabolic pathways, regardless of the expression or flux activity of individual genes. This approach helps to mitigate the effects of gene expression variability and ensures that the core reaction sets generated are biologically relevant. Our results demonstrate the potential of our method to provide a deeper understanding of the impact of thresholding on the accuracy of context-specific models. Our findings are important for advancing the field, as they shed light on how ssGSEA can be utilized to extract biologically relevant information from gene expression data.

We acknowledge that our method has limitations and that there is room for improvement. However, we are confident that our results provide a solid foundation for future work in this area and represent a step forward in developing context-specific models.

## Materials and methods

### An overview of the conceptual framework for transcriptional data integration into a GEM

To devise and validate our new framework for integrating transcriptomics data into a GEM, we have combined two previously developed algorithms (ssGSEA and GIMME) to increase the accuracy of metabolic functionalities of GEMs.

### ssGSEA, a gene set enrichment analysis approach

Gene Set Enrichment analysis (GSEA) is a functional genomic analysis, which uses the pre-defined set of genes/proteins across high-throughput data and determines statistically significant sets that are concordantly different between two biological states. Indeed, this method is an approach for finding over-represented genes associated with a phenotype. GSEA identifies either significantly enriched (top of ranked genes list) or depleted (bottom of ranked genes list) gene sets and elicits a quantitative enrichment score of over-represented gene sets at the top or bottom of the list of the ranked genes. Then, this method uses the permutation test to estimate *P*-values and finally normalizes the enrichment score (NES) for each gene set and adjusted *P*-value to multiple hypotheses testing and False Discovery Rate (FDR) presented. Single sample Gene Set Enrichment analysis (ssGSEA)^21^ is a customized version of GSEA, that similar to GSEA detects biologically relevant gene sets that are over-represented (top or bottom of the ranked gene) in a single sample. The ssGSEA calculates an ES in a single sample without requiring control data. The genes for a sample were ranked (and normalized) from high to low using the Empirical Cumulative Distribution Functions (ECDF) and absolute expression values. The ES is obtained by a sum (integration) of the differences between the weighted ECDF among genes of a gene set relative to the genes that are not existent in the given set.

### GIMME, an algorithm for creating a consistent GEM with a desired metabolic objective

The GIMME algorithm^9^ weights the reactions by gene expression values and uses a threshold for minimizing low-expressed reactions as inactive while keeping the objective above a certain value. In this study, we used the adapted version of the original GIMME by S. Opdam and A. Richelle 2017^7^, in which differences in threshold and expression levels were used as weights and set as objective coefficients of the reactions. The threshold was 25 percent quantile subtracted from the expression data and reactions without expression data were given a weight of −1, which prevented those reactions from deletion during model construction. We set the tolerance for the reduction of the value of the objective function to 0.9.

### ssGSEA-GIMME, a combined framework for data normalization and integration

To improve the accuracy of integration methods, we combined the results of ssGSEA and the GIMME methods. To categorize reactions of the yeast metabolic model with high confidence, we mapped metabolic processes to the model gene-protein-reaction (GPR) associations, using a manually curated annotation set of proteins, assigned to different metabolic processes, first introduced in ref. ^22^. The ssGSEA analysis was performed using R code provided by Broad institute (Original code written by Pablo Tamayo and modified by Karsten Krug) (https://github.com/broadinstitute/ssGSEA2.0). The minimum number of genes needed for a gene set was three. We used the area under curve (“area.under.RES”) as a statistic, the z-score as correlation type, and performed 1000 permutations. In our analysis, we used a z-score cut-off value of 1.96. The scores calculated by the ssGSEA analysis were used as weights to map genes expression values to the corresponding reaction expression values, which we then fed into GIMME to construct the context specific GEM.

### The genome-scale metabolic model of yeast

We used the consensus genome-scale metabolic model of *S. cerevisiae*. Version 8.4.2 was downloaded from the project’s GitHub repository (https://github.com/SysBioChalmers/yeast-GEM), which contains 4058 reactions, 2742 metabolites, and 1150 genes.

All bounds used the original flux bounds of the model, except for the experimentally determined fluxes through ‘D-glucose exchange’ (as measured) and ‘oxygen exchange’ (setting the lower bound to maximal determined uptake rate) for the first study (glucose-limited chemostats), and ‘D-glucose exchange’, ‘ethanol exchange’, ‘acetate exchange’, and ‘maltose exchange’ for the second study (carbon source-limited chemostats). We set the biomass function to ‘biomass pseudoreaction’ for both studies.

### Omics data collection; RNA-seq and flux data

We collected the omics data (transcriptome) and physiological measurements from two studies. First, in an unpublished dataset provided by Simon Hubbard (University of Manchester, UK), *S. cerevisiae* strain CEN.PK113-7D was grown in a chemically-defined minimal (Verduyn) medium with glucose as the main carbon source. Cells were cultivated in two independent chemostats at dilution rates spanning from $D=0.20{h}^{-1}$ to $0.34{h}^{-1}$ and both supernatant and cell samples were taken for determination of exometabolite fluxes^22^, using analytical procedures described in^23^ and RNA-seq analysis, as described in^24^. In this study, the sequencing was performed on the ABI SOLiD platform. The reads were aligned to the *S. cerevisiae* genome assembly EF4, which was downloaded from ENSEMBL, using the Bowtie version 1 software^25^. To determine the expression values, the Reads Per Million (RPM) normalization method was applied.

In a study by Daran-Lapujade et al.^14^, *S. cerevisiae* strain CEN.PK113-7D was grown in a chemically-defined minimal (Verduyn) medium with one of four carbon sources (glucose, maltose, ethanol, or acetate) as the main carbon source at $D=0.10{h}^{-1}$. The results of transcriptomics and flux analysis were described in^14^. Microarray profiling data was taken from the GEO database, accession number GSE8895. The Microarray Analysis consisted of taking samples from chemostats, preparing the probes, and hybridizing them with Affymetrix GeneChip® microarrays. The findings for each growth scenario were derived from three separate, independently grown replicates.

### Generation of context-specific GEMs

For each condition, we created two sets of context-specific GEMs: the first one constructed by deploying the standard GIMME method and the second one, deploying GIMME with transcriptomics data, enriched by ssGSEA. All models and related codes are available from the GitHub repository https://github.com/mahjalili/ssGSEAGEM.

## Supplementary information

Figure S1.docx

nr-reporting-summary

## Data Availability

The experimental data from glucose-limited chemostat studies are partially available: RNA-seq data was provided by Prof. Simon Hubbard and therefore not made public in this manuscript and flux data is reported in (10.1101/2021.06.11.448029v2). Data from different carbon source-limited chemostats is publicly available (10.1074/jbc.M309578200).
