# Supplementary material for: Metabolic function-based normalization improves transcriptome data-driven reduction of genome-scale metabolic models
Source: NPJ Syst Biol Appl. 2023 May 20;9:15. doi: 10.1038/s41540-023-00281-w (PMC10199931; doi:10.1038/s41540-023-00281-w)
Supplement: Supplementary file 1 — Figure S1.docx [file 41540_2023_281_MOESM1_ESM.docx]

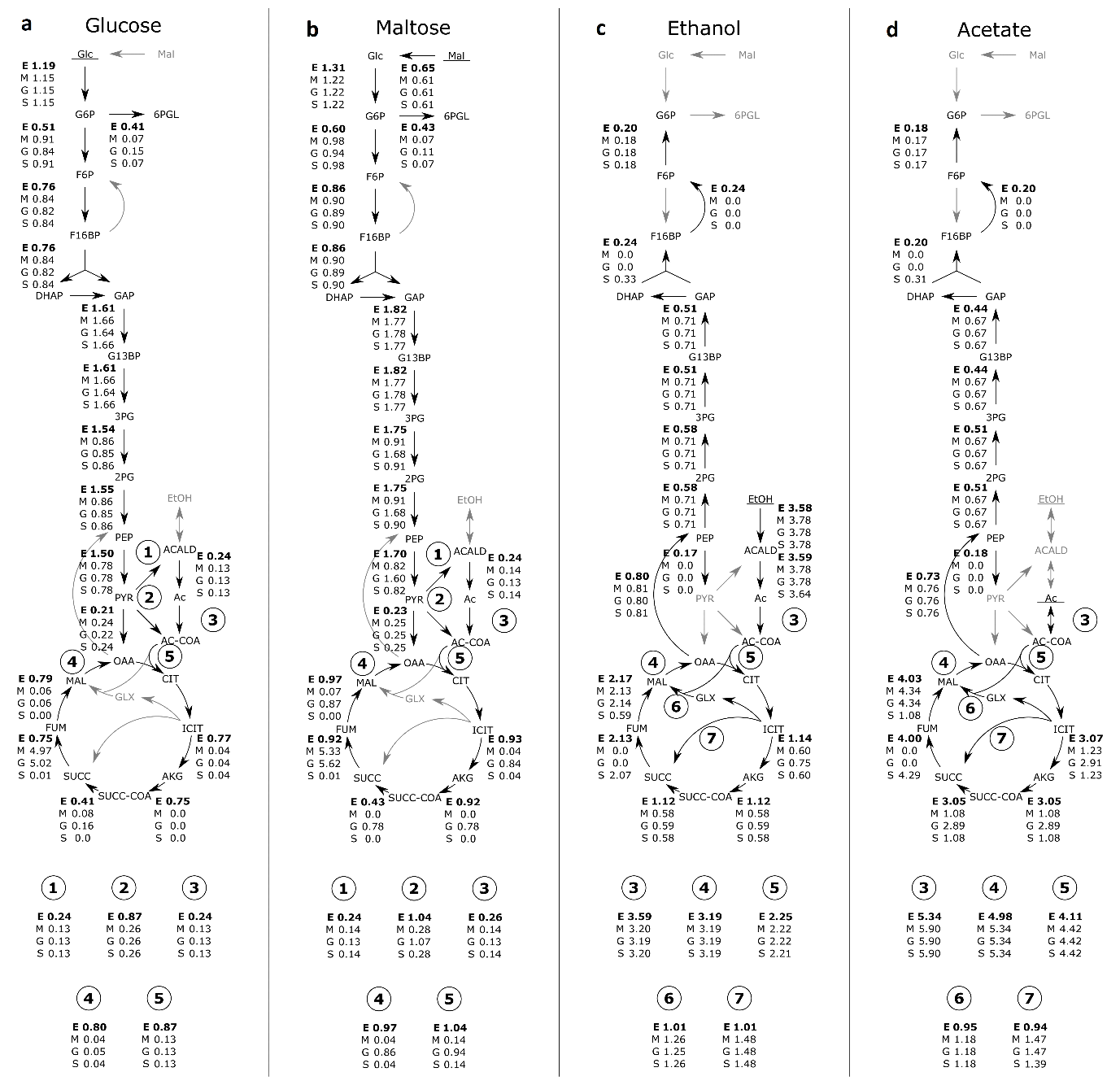


**Figure S1.** Experimentally determined (E) and predicted fluxes (in ${{mmol}/{gDW}}/h$) by the conventional GEM (M), GIMME (G) and ssGSEA-GIMME (S) in central carbon metabolism for carbon source-limited growth of *S. cerevisiae* at $D=0.1h^{-1}$. Metabolites and reactions in gray represent interconversions, which do not happen during growth on these carbon sources. Points-of-entry of these carbon sources to the metabolic network are marked with a bar under the respective abbreviation. For abbreviations, see the caption of Figure 1.
